# Inhibitory effects of pH, salinity, and tea polyphenols concentration on the specific spoilage organisms isolated from lightly‐salted large yellow croaker (*Pseudosciaena crocea*)

**DOI:** 10.1002/fsn3.2905

**Published:** 2022-05-08

**Authors:** Quan‐you Guo, Ke Shan, Xu Yang, Chao‐jun Jiang, Lin Zhu

**Affiliations:** ^1^ East China Sea Fisheries Research Institute，Chinese Academy of Fishery Sciences Shanghai China; ^2^ University of Shanghai for Science and Technology Shanghai China

**Keywords:** antibacterial effect, growth dynamic, *Hafnia alvei*, lightly‐salted *Pseudosciaena crocea*, *Proteus vulgaris*

## Abstract

*Proteus vulgaris* and *Hafnia alvei* were identified as specific spoilage organisms (SSOs) isolated from the refrigerated lightly‐salted large yellow croaker (*Pseudosciaena crocea*). In this work, the inhibitory effects of pH, salinity, and tea polyphenols concentration on both strains were investigated. Modified Gompertz models were used to estimate the kinetic parameters *μ_m_* (maximum specific growth rate) and *λ* (duration of lag phase) of the two strains under different conditions, demonstrating that their growth rates decreased with the decrease of pH as well as the increase of salinity and tea polyphenols concentration, and the growths of both strains stopped while the salinity and tea polyphenols concentration increased to 0.05 and 5%, respectively. Response surface methodology (RSM) based on a three‐level three‐factor Box–Behnken Design (BBD) was employed to optimize the combination of these three antibacterial factors. The results showed that the optimum inhibitory conditions were: tea polyphenols concentration 0.05%, salinity 3.46%, and pH 6.96 to inhibit the growth of *P*. *vulgaris*; tea polyphenols concentration 0.05%, salinity 3.45%, and pH 6.94 to inhibit *H*. *alvei*. Validation experiments were performed and demonstrated that under these conditions, the growth of the two SSOs could be 100% inhibited. This research provided references for the inhibition of the SSOs of lightly‐salted large yellow croaker and the extension of its shelf life.

## Graphical abstract

*Proteus vulgaris* and *Hafnia alvei* were identified as specific spoilage organisms (SSOs) isolated from the refrigerated lightly‐salted large yellow croaker (*Pseudosciaena crocea*). In this work, the inhibitory effects of pH, salinity, and tea polyphenols concentration on both strains were investigated. The results showed that the optimum inhibitory conditions were: tea polyphenols concentration 0.05%, salinity 3.46%, and pH 6.96 to inhibit the growth of *P*. *vulgaris*; tea polyphenols concentration 0.05%, salinity 3.45%, and pH 6.94 to inhibit *H*. *alvei*.

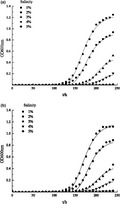

## 1 INTRODUCTION

Large yellow croaker (*Pseudosciaena crocea*) is an important economic fish in the coastal waters of China. It is distributed in the southern Yellow Sea, the East China Sea, the Taiwan Strait, and the South China Sea east to the Leizhou Peninsula, featuring with tender texture and rich nutrition (Yang et al., 2014). Salting is a traditional way to process large yellow croaker. With the accelerating pace of life, convenient and healthy food become a consumer trend, which encourages the development of minimally processed food with low salt. However, under such conditions as low salt and high moisture, large yellow croaker is vulnerable to spoilage which shortens its shelf life. Microorganisms are the main factors causing the spoilage of aquatic products. One or several major microorganisms responsible for the spoilage of a given product were defined as specific spoilage organisms (SSOs) (Gram & Huss, 1996). Previous research showed that *Proteus vulgaris* and *Hafnia alvei* were the SSOs for the lightly‐salted large yellow croaker stored at 5℃ (Guo et al., 2017). It has become a research hotspot in the field of aquatic products preservation to develop effective methods to inhibit or eliminate the SSOs and prevent spoilage (Gram & Dalgaard, 2002).

Hurdle technology can inhibit the growth of SSOs while minimizing the processing of the products, by putting microorganisms under multiple stress factors including low temperature, low water activity (Aw), acidity, or so on (Leistner & Gorris, 1995). Hurdle technology has been applied in the preservation of many aquatic products, such as dolphinfish (*Coryphaena hippurus* Linnaeus) (Messina et al., 2015), hairtail (Hu et al., 2014), ready‐to‐eat shrimp (Kanatt et al., 2006), and oyster (Chen et al., 2010). Guo et al. (2018) examined the inhibitory effect of hurdle factors on the growth of *Vibrio alginolyticus* in lightly‐salted large yellow croaker, finding salinity and pH not enough to inhibit the growth of SSOs and biological preservatives necessary. Tea polyphenols, a type of biological preservatives which are natural, antibacterial, antioxidative, and antiviral, have been widely used in food preservation. Research shows that tea polyphenols can effectively inhibit the growth of SSOs in aquatic products (Wang, 2013). Zhang et al. (2011) found that 0.2% tea polyphenols could prolong the shelf life of large yellow croaker for 7–8 days at 4℃. It is a promising preservative to inhibit the SSOs in lightly‐salted yellow croaker along with salinity and pH.

Predictive microbiology effectively combines mathematical model, microbiology, and computer technology to quantitatively evaluate the growth, death and, dormancy of microorganisms. Zhou et al. (2015) used first‐order and second‐order kinetic models to describe the growth of *Listeria monocytogenes* in raw fish fillets. Vermeulen et al. (2007) developed a growth/nongrowth interface model to describe inhibitory effect of low temperature, pH, Aw, and acetic acid on the growth of *Listeria* spp. Response surface methodology (RSM) can simultaneously compare and optimize multiple factors and their interactions and obtain the optimal level of each factor. Jiang et al. used RSM to optimize the factors to inhibit the growth of *L*. *monocytogenes* in salmon for better preservation (Jiang et al., 2017).

The objective of this work was to investigate the inhibitory effects of pH, salinity, and tea polyphenols concentration separately on the two SSOs *P*. *vulgaris* and *H*. *alvei* isolated from lightly‐salted large yellow croaker, by developing growth models to estimate and compare the kinetic parameters of the two strains under different hurdle factors stress. RSM was then used to optimize the combination of these three hurdle factors to inhibit the growth of the SSOs, which can provide a reference for extending the shelf life and improving the quality of light‐salted large yellow croaker.

## 2 MATERIALS AND METHODS

### 2.1 Main reagents and instruments

Nutritional agar (AR), nutrient broth (BR), sodium chloride (AR), Shanghai Sinopharm Chemical Reagent Co., Ltd; Tryptone Soybean Broth (TSB) (pH 7.3 + 0.2, 0.5% NaCl), Shanghai Zhongke Insect Biotechnology Development Co., Ltd; HCL Standard Solution: 6 mol/L, Shenzhen Bolinda Technology Co., Ltd; Tea polyphenol, Wuhan Baixing Biotechnology Co., Ltd; Microtiter plate, Finland Bioscreen Co., Ltd.

pH Meter: pHS‐3C, Shanghai Leici Instrument Factory; Microbial Growth Meter: Bioscreen C, Finland Bioscreen Company; Super Clean Workbench: SW‐CJ‐1FB, Shanghai Boxun Medical Equipment Factory; High Precision Low Temperature incubator: MIR‐153, Shanghai Yiheng Science Instrument Co., Ltd; Vortex Mixer: QT‐2, Shanghai Qite Analytical Instrument Co., Ltd; Fully Automatic Stainless Steel High Temperature Steam Cooker: ZM‐100, Guangzhou Standard International Packaging Equipment Co., Ltd; Ultra‐low Temperature Preservation Box: DW‐86L626, Qingdao Haier Special Electrical Appliances Co., Ltd; Refrigerator: BD/BC‐288, Fuzhou Fuxue Island Refrigeration Equipment Co., Ltd.

### 2.2 Preparation of bacterial suspension samples

Lightly‐salted large yellow croaker was processed by a fishery company in Ningde city, Zhejiang province, China by back‐cutting, cleaning, salting, drying, and vacuum packaging. After being transported to Shanghai Laboratory by refrigerated truck, it was frozen at −18℃ for reservation. Product characteristics: salinity: (2.0 ± 0.12)%, water content: (60.79 ± 2.24)%, water activity: 0.96 ± 0.002.

The SSOs of lightly‐salted large yellow croaker were identified as *P*. *vulgaris* (serial number: KY684257) and *H*. *alvei* (serial number: KY684258), with the proportion of 58.9% and 35.9%, respectively, by MIDI gas chromatography and 16S rRNA sequencing. The strains were isolated and preserved at −80℃ (Guo et al., 2017).

*Proteus vulgaris* and *H*. *alvei* preserved at ultra‐low temperature were inoculated into sterile nutrient broth, respectively, shaken evenly and cultured in a constant temperature incubator for 24 h at 25℃, and isolated to get single colonies by streaking. The isolated strain was inoculated into 10 ml sterile TSB and cultured at 25℃ for 24 h until the concentration of bacterial suspension reached 10^8^ CFU/ml. Finally, the suspension was diluted to 10^4^ CFU/ml by gradient of sterile saline.

### 2.3 Experimental design

Because of the low salinity and weak acidity of lightly‐salted large yellow croaker, three factors (pH, salinity, and tea polyphenols concentration) were investigated with five levels for each factor. Based on the results of preexperiment, the salinity was set as 1, 2, 3, 4, and 5% with a pH of 7.0; pH was adjusted by HCI to 5.0, 5.5, 6.0, 6.5, and 7.0 with salinity at 0.5%; tea polyphenols concentration was set as 0.01, 0.03, 0.05, 0.07, and 0.09% with pH at 7.0 and salinity at 0.5%. The corresponding TSB inoculation solutions were prepared accordingly and sterilized at 121℃ for 15 min.

The prepared sterile inoculation solutions were added to the sterile 96‐well microtiter plate as 180 μl per well, with 20 μl 10^4^ CFU/ml bacterial suspension. Four replicates were made for each condition and sterile TSB broth (salinity 0.5%, pH 7.0) was used as control. The microtiter plate was then incubated in Bioscreen, a microbial growth analyzer, at 5℃ for 10 days, and the optical density at 600 nm (OD_600_) of the content of each well was measured and recorded every hour.

### 2.4 Modeling

#### 2.4.1 Development of the growth kinetics models

The modified Gompertz model was used to describe the growth of two strains in different conditions and estimate the kinetic parameters (Zweitering & Jongenburger, 1990):

(1) $$N_{t}=N_{0}+\left(N_{\max}‐N_{0}\right)\times \exp\left\{‐\exp\left[\frac{e*\mu_{m}}{\left(N_{\max}‐N_{0}\right)}\left(\lambda ‐t\right)+1\right]\right\}$$

where *t* is time (in h); $N_{t}$ is OD_600_ at time t (in absorbance units); $N_{\max}$ is maximum OD_600_ (in absorbance units); $N_{0}$ is initial OD_600_ (in absorbance units); $\mu_{m}$ is maximum specific growth rate (in absorbance units h^‐1^); *λ* is the duration of lag phase (in h). The experimental data were fitted with Origin 9 (American Origin Lab Co. Ltd) and SPSS 19.0 (American IBM Co. Ltd).

The goodness‐of‐fit of the models were evaluated by determinant coefficient *R* ^2^, accuracy *A_f_*, deviation *B_f_*, and RMS, calculated as follows:

(2) $$A_{f}=10\frac{\sum \left|\log\left(X_{\mathrm{cal}}/X_{\mathrm{obs}}\right)\right|}{n}$$

(3) $$B_{f}=10\frac{\sum \log\left(X_{\mathrm{cal}}/X_{\mathrm{obs}}\right)}{n}$$

(4) $$\mathrm{RMS}=\sqrt{\frac{\sum {\left(X_{\mathrm{cal}}/X_{\mathrm{obs}}\right)}^{2}}{n}}$$

where *X*_cal_ is the predicted value and *X*_obs_ is the measured value. The closer the *R* ^2^, *A_f_*, and *B_f_* values are to 1, or the closer the RMS is to 0, the better the prediction is.

#### 2.4.2 Response surface methodology

Based on the results of single inhibitory factors, an RSM was used to optimize the combination of three factors with Box–Behnken Design. The experiment was designed and data were analyzed by Design‐Expert 8.0.6 software with 3 levels for each factor (Table 1) and 17 different conditions were yielded. Each condition was performed in quadruplicate, and TSB broth (pH 7.3 + 0.2, NaCl 0.5%) was used as blank control group.

**TABLE 1:** Three factors and three levels used in the Box–Behnken design

Levels	Factors		
	Tea polyphenol concentration (%)	Salinity (%)	pH
−1	0.01	1	5.0
0	0.03	3	6.0
1	0.05	5	7.0

The inhibitory rate was used as response value to evaluate the inhibitory effect of each condition, calculated as follows (Yang et al., 2012):

(5) $$\text{Inhibitory}\;\text{rate}\left(\%\right)=\frac{{\text{OD}}_{600\text{nm}}\text{control}‐{\text{OD}}_{600\text{nm}}\text{experimental}}{OD_{600\text{nm}}\text{control}}\times 100\%$$

## 3 RESULTS AND DISCUSSION

### 3.1 Inhibitory effects of pH, salinity, and tea polyphenols (TPs) concentration on the growth of the SSO strains

#### 3.1.1 Inhibitory effect of tea polyphenols concentration

Tea polyphenols (TPs) have broad‐spectrum inhibitory effects on pathogenic microorganisms by suppressing the critical steps of their pathogenic processes. TPs can damage the cell wall of bacteria and increase the permeability of their cell membrane, leaking the contents of cells and leading them to metabolic disorder (Sun & Wang, 2009).

**FIGURE 1:**
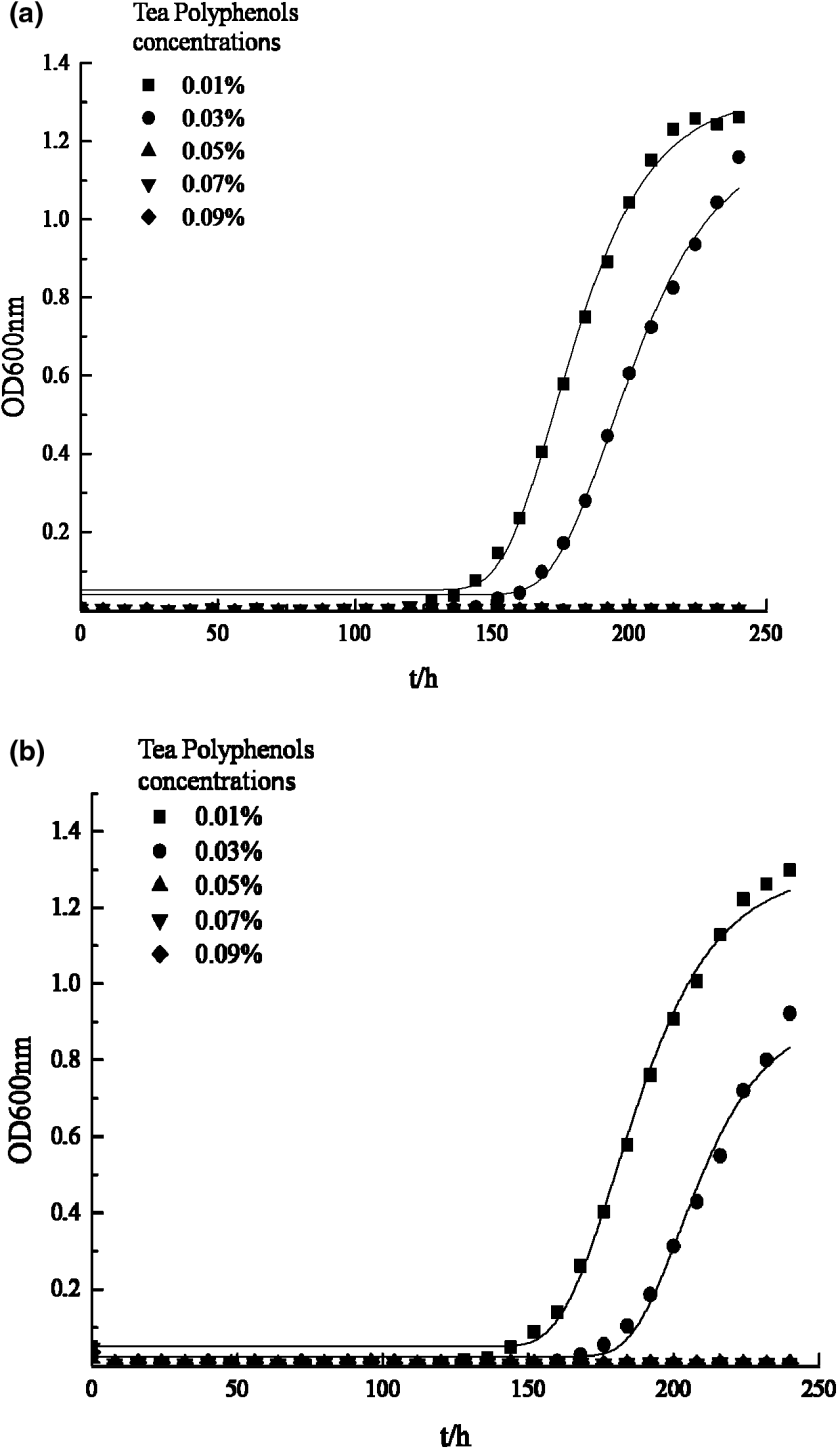
Effect of different tea polyphenol concentrations on the growth of SSOs. (a) The growth curve of *Proteus vulgaris* at different tea polyphenol concentration; (b) The growth curve of *Hafnia alvei* at different tea polyphenol concentration

**FIGURE 2:**
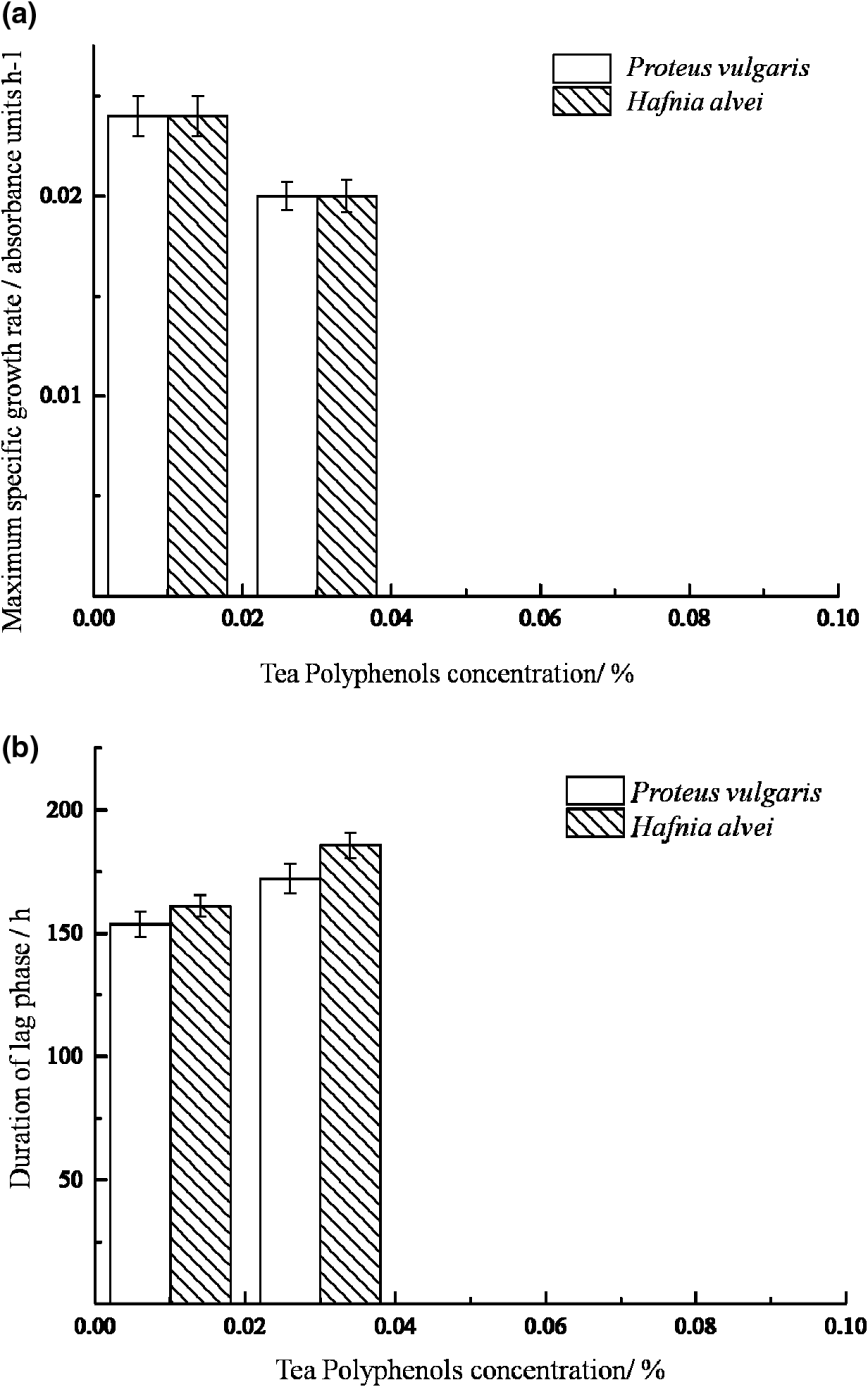
Growth kinetics of the SSOs at different tea polyphenol concentrations. (a) Maximum specific growth rate of two SSOs at different tea polyphenol concentration; (b) Duration of lag phase of two SSOs at different tea polyphenol concentration

The growth of *P*. *vulgaris* and *H*. *alvei* under different TPs concentrations are shown in Figure 1. The growth curves are well fitted with modified Gompertz model, as the correlation coefficients *R* ^2^ were greater than 0.980, while Afs were between 1.000 and 1.05, Bfs were equal to 1.000, and RMS were between 0.000 and 0.050. Figure 2 illustrates the effect of TPs on $\mu_{m}$ and *λ* of two bacteria strains. With the increase of tea polyphenols concentration, $\mu_{m}$ decreased while *λ* increased, indicating that tea polyphenols have good inhibitory effect on the two bacteria. When the TPs concentration increased to 0.05%, neither of the two bacteria grew, indicating that a growth/nongrowth point of the two bacteria could be found under the effect of TPs between 0.03% and 0.05% concentration according to Xiu's method (Xiu et al., 2016). This result is consistent with previous researches which showed that TPs have efficient antibacterial effects in vitro and in vivo, and their minimum inhibitory concentration (MIC) on several common microorganisms in food (such as *Staphylococcus aureus*, *Salmonella*, *Bacillus subtilis*, etc.) are all <0.1% (Li et al., 2021).

#### 3.1.2 Inhibitory effect of salinity concentration

**FIGURE 3:**
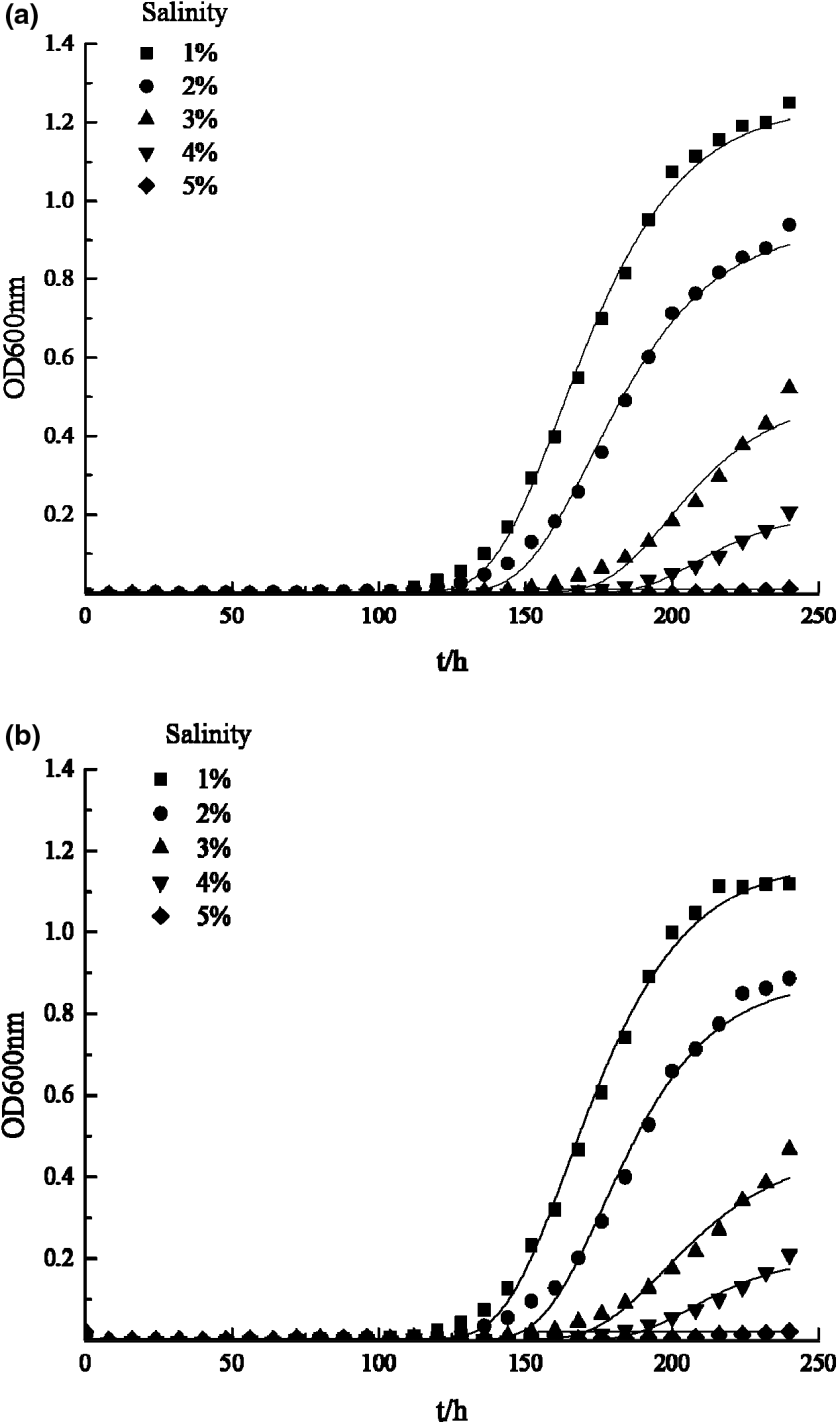
Effect of different salinity on the growth of SSOs. (a) The growth curve of *Proteus vulgaris* at different salinity; (b) The growth curve of *Hafnia alvei* at different salinity

**FIGURE 4:**
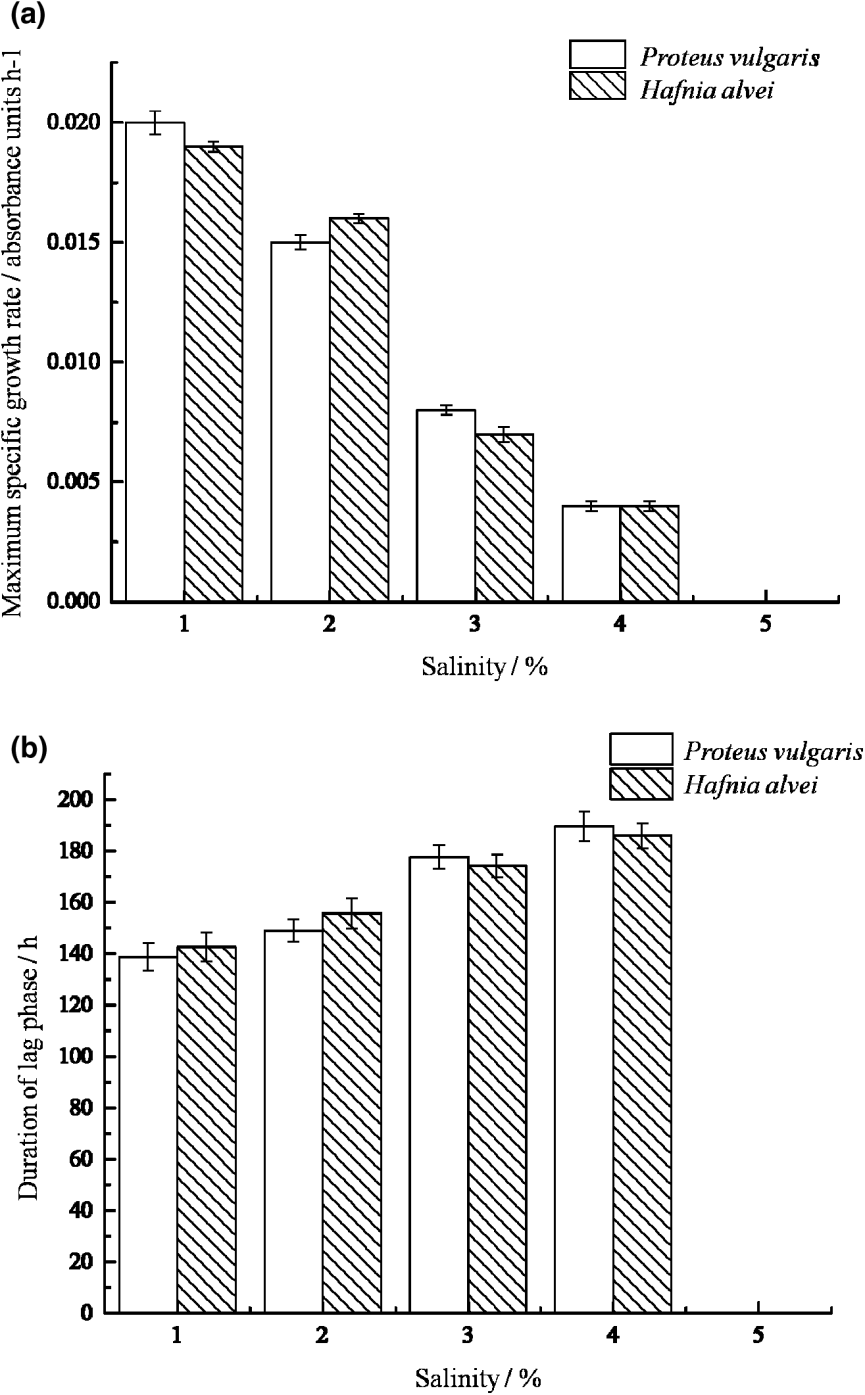
Growth kinetics of the SSOs at different salinity. (a) Maximum specific growth rate of two SSOs at different salinity; (b) Duration of lag phase of two SSOs at different salinity

Salinity is one of the most common environmental hurdle factors. Na^+^ can separate the cytoplasm and cell wall by creating hyperosmotic environment, inhibit the synthesis of macromolecule substances, and thus inhibit cell growth until death (Vyrides & Stuckey, 2009). Also, microorganisms have to consume energy to produce extracellular polymers and other substances to maintain equilibrium when the ion concentration in solution increases and will die when the pressure is too high (Blight & Ralph, 2004). The effects of salinity on growth and kinetic parameters of two bacteria strains are shown in Figures 3 and 4. The growth curves are well fitted with modified Gompertz model, as the correlation coefficients R^2^ were greater than 0.9870, while Afs were between 1.000 and 1.060, Bfs were between 1.000 and 1.050, and RMS were between 0.000 and 0.070. The figures indicate that with the increase of salinity, $\mu_{m}$ decreased while *λ* increased, demonstrating salinity has good inhibitory effect on the two bacteria. When the salinity increased to 5%, neither of the two bacteria grew. This result is consistent with other research (Olajide & Ogbeifun, 2010). The bacteria can secrete signaling molecules such as AHLs for communication to promote the growth, which is described as quorum sensing. Kong et al. (2017) found that the signaling molecules of *H*. *alvei* was most active at 2% salinity, and decreased with the increase of salinity. This can explain the inhibitory effect of salinity on the growth of the bacteria on the other hand.

#### 3.1.3 Effects of pH on the growth kinetics and model evaluation

**FIGURE 5:**
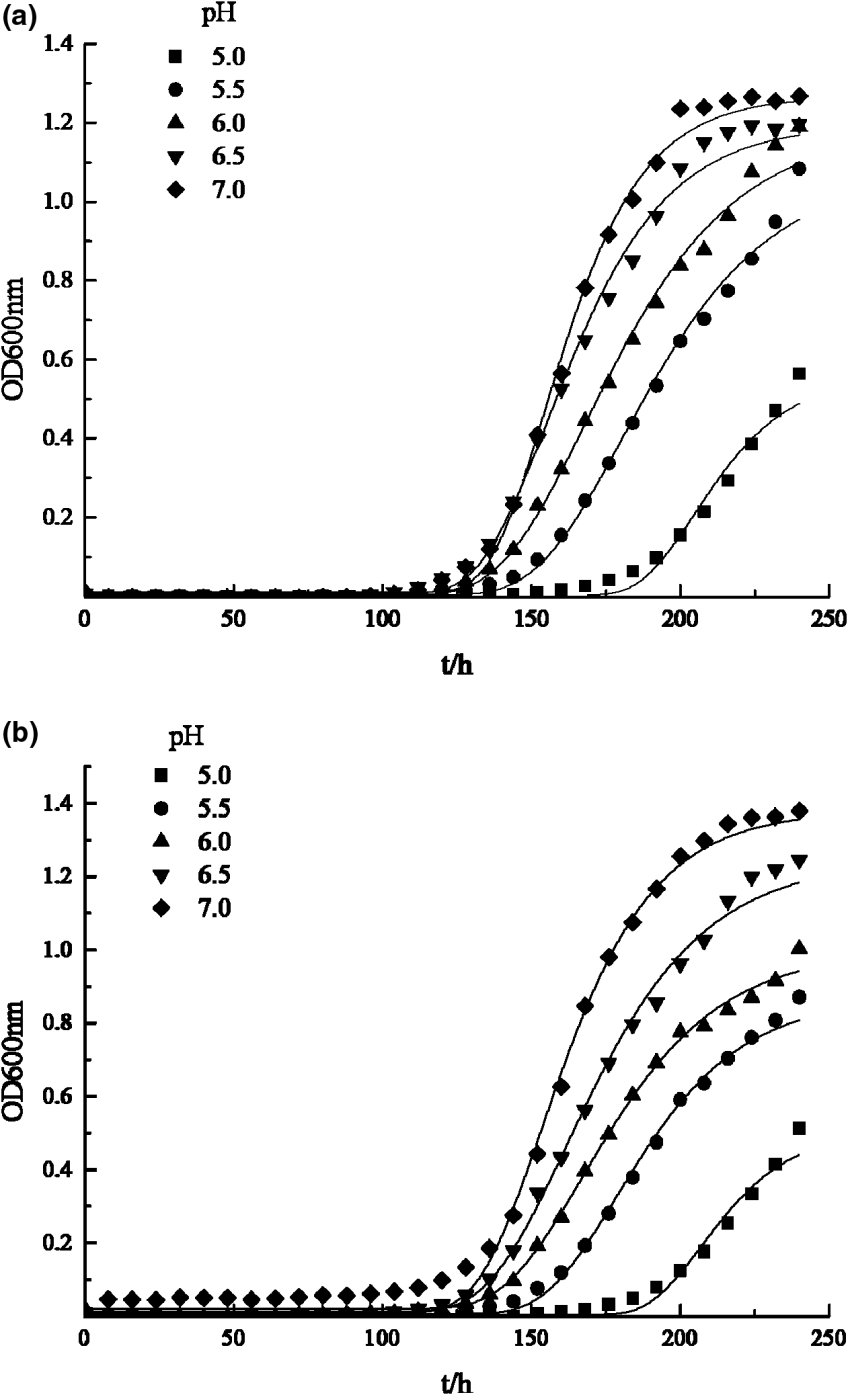
Effect of different pH on the growth of SSOs. (a) The growth curve of *Proteus vulgaris* at different pH; (b) The growth curve of *Hafnia alvei* at different pH

**FIGURE 6:**
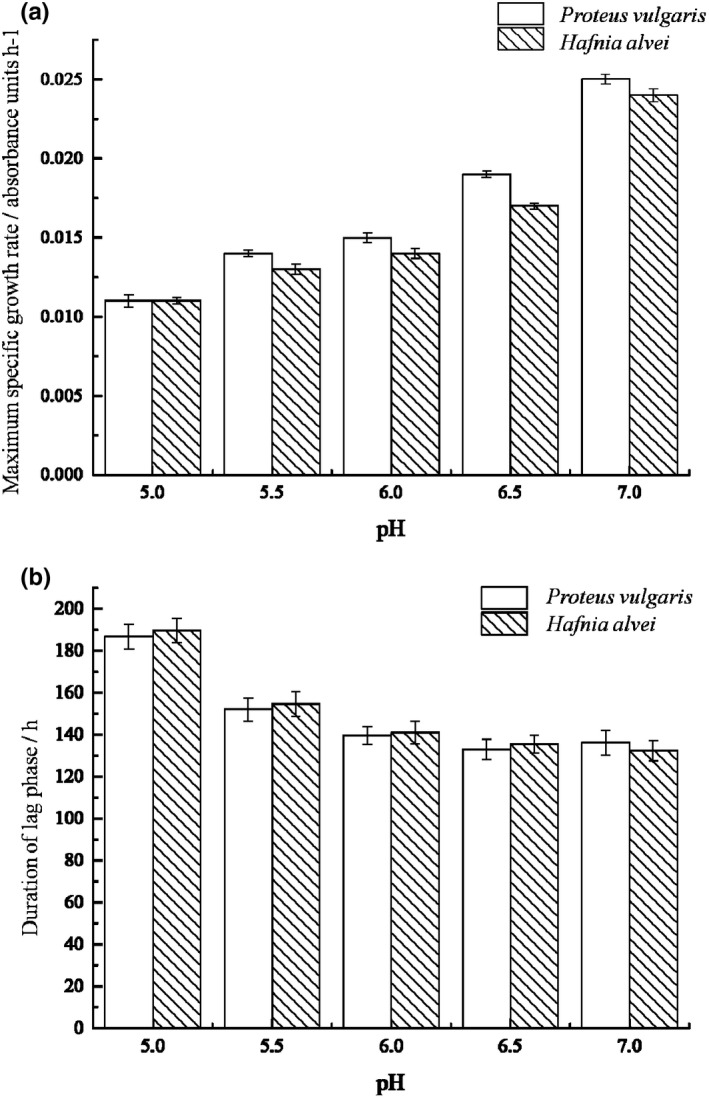
Growth kinetics of the SSOs at different pH. (a) Maximum specific growth rate of two SSOs at different pH; (b) Duration of lag phase of two SSOs at different pH

pH can affect cell membrane permeability, biofilm formation, bacterial surface ultrastructure, and bacterial metabolism (Dai, 2016; Xiu et al., 2016). The effect of pH on the growth curve and kinetic parameters of the two strains are showed in Figures 5 and 6. The growth curves are well fitted with modified Gompertz model, as the correlation coefficients *R* ^2^ were greater than 0.980, while Afs were between 1.000 and 1.080, Bfs were between 1.000 and 1.100, and RMS were between 0.000 and 0.100. The figures indicate that with the increase of pH, $\mu_{m}$ increased continuously. On the other hand, *λ* decreased when pH increased from 5.0 to 6.0, and kept stable when pH increased from 6.0 to 7.0.

These results coincided with the study which showed *P*. *vulgaris* could grow better in neutral and weak alkaline environment (Oladipo & Adejumobi, 2010). The result of *H*. *alvei* was similar to that of *P*. *vulgaris*. The growth of *H*. *alvei* in different pH may be related to the formation of biofilm. Ma et al. (2017) found that the change of pH could affect the formation of biofilm of *H*. *alvei*, and the biofilm formation was the highest at pH 7.0. The pH of light‐salted large yellow croaker is between 5.8 and 7.0. Within this pH range, *H*. *alvei* and *P*. *vulgaris* can maintain growth, making them to eventually become the specific spoilage bacteria in the final products.

### 3.2 Response surface model and verification

#### 3.2.1 Response surface model and significance analysis

On the basis of single factor experiment, response surface optimization and analysis were designed according to Box–Behnken principle, and the results were analyzed by software (Table 2). The predictive models were:

$$\begin{array}{c} \mathrm{Pr}oteus\;vu\mathrm{lg}aris‐\text{inhibitory rate}\left(\%\right)=723.99‐5907.5*A+35.36\\ {}*B‐221.85*C‐81.25*A*B+875.00*A*C\\ +22,625.00*A^{2}‐1.55*B^{2}+16.05*C^{2}; \end{array}$$

$$\begin{array}{c} Hafnia\;alvei‐\text{inhibitory rate(\textbackslash{}\% )}=679.01‐5836.25\\ {}*A+43.08*B‐209.65*C+12.50*A*B\\ +700.00*A*C+0.63*B*C+33,312.5\\ {}*A^{2}‐3.86*\;B^{2}+15.08*\;C^{2}. \end{array}$$

where *A* is the tea polyphenols concentration, *B* is salinity, and *C* is pH.

**TABLE 2:** Experimental design and results of the response surface methodology

No.	Hurdle factors			Inhibitory rate (%)	
	A: Tea polyphenol concentration (%)	B: Salinity (%)	C: pH	*Proteus vulgaris*	*Hafnia alvei*
1	0.01	5	6.0	99	97
2	0.03	1	5.0	20	5
3	0.01	3	7.0	34	39
4	0.03	5	7.0	97	100
5	0.05	3	7.0	100	100
6	0.01	1	6.0	0	3
7	0.03	1	7.0	20	0
8	0.01	3	5.0	84	88
9	0.05	5	6.0	98	97
10	0.03	3	6.0	51	47
11	0.03	3	6.0	42	49
12	0.03	5	5.0	100	100
13	0.03	3	6.0	49	62
14	0.03	3	6.0	60	54
15	0.03	3	6.0	45	46
16	0.05	3	5.0	80	93
17	0.05	1	6.0	12	1

Table 3 shows the result of variance analysis of the fitting equation of *P*. *vulgaris* and *H*. *alvei*. *P*‐value of the two regression models is <0.01, and the lack of fit is not significant (>.05). The correlation coefficients *R^2^* were 96.30% and 96.09% respectively, and the revised correlation coefficients $R_{\text{adj}}^{2}$ were 91.53% and 91.05%, respectively, indicating that the model fitted well and could be used to analyze and predict the inhibitory effect of the combination of three factors on *P*. *vulgaris* and *H*. *alvei*.

**TABLE 3:** Variance analysis results of the developed response surface models

Source of variance	*Proteus vulgaris*					*Hafnia alvei*				
	Sum of squares	Degree of freedom	Mean square	F	P	Sum of squares	Degree of freedom	Mean square	*F*	*p*
Model	1.83	9	0.20	20.22	0.0003	2.27	9	0.25	19.09	0.0004
A	0.07	1	0.07	6.63	0.0368	0.05	1	0.05	3.87	0.0898
B	1.46	1	1.46	145.49	<0.0001	1.85	1	1.85	140.14	<0.0001
C	0.01	1	0.01	1.35	0.2826	0.03	1	0.03	2.09	0.1916
AB	4.225E−003	1	4.225E−003	0.42	0.5374	1.000E−004	1	1.000E−004	7.564E−003	0.9331
AC	0.12	1	0.12	12.19	0.0101	0.08	1	0.08	5.93	0.0451
BC	2.250E−004	1	2.250E−004	0.02	0.8853	6.250E−004	1	6.250E−004	0.05	0.8341
A^2^	0.03	1	0.03	3.43	0.1064	0.08	1	0.08	5.65	0.0490
B^2^	0.02	1	0.02	1.61	0.2450	0.1	1	0.10	7.58	0.0284
C^2^	0.11	1	0.11	10.79	0.0134	0.10	1	0.10	7.24	0.0311
Residual	0.070	7	0.010			0.093	7	0.013		
Lack of fit	0.051	3	0.017	3.62	0.1228	0.075	3	0.025	5.79	0.0614
Pure error	0.019	4	4.730E−003			0.017	4	4.330E−003		
Sum	1.90	16				2.36	16			

The results of coefficient significance test showed that salinity (*B*) had very significant (*p* < .01) inhibitory effect on both strains, tea polyphenols concentration (*A*) had significant (*p* < .05) inhibitory effect, while pH (*C*) could not effectively inhibit their growth; *C* ^2^ had significant inhibitory effect while *B* ^2^ and *C* ^2^ had no significant effect; the interaction between *A* and *C* played important role on the inhibitory effect, while other two interactions were not significant.

#### 3.2.2 Interaction analysis

**FIGURE 7:**
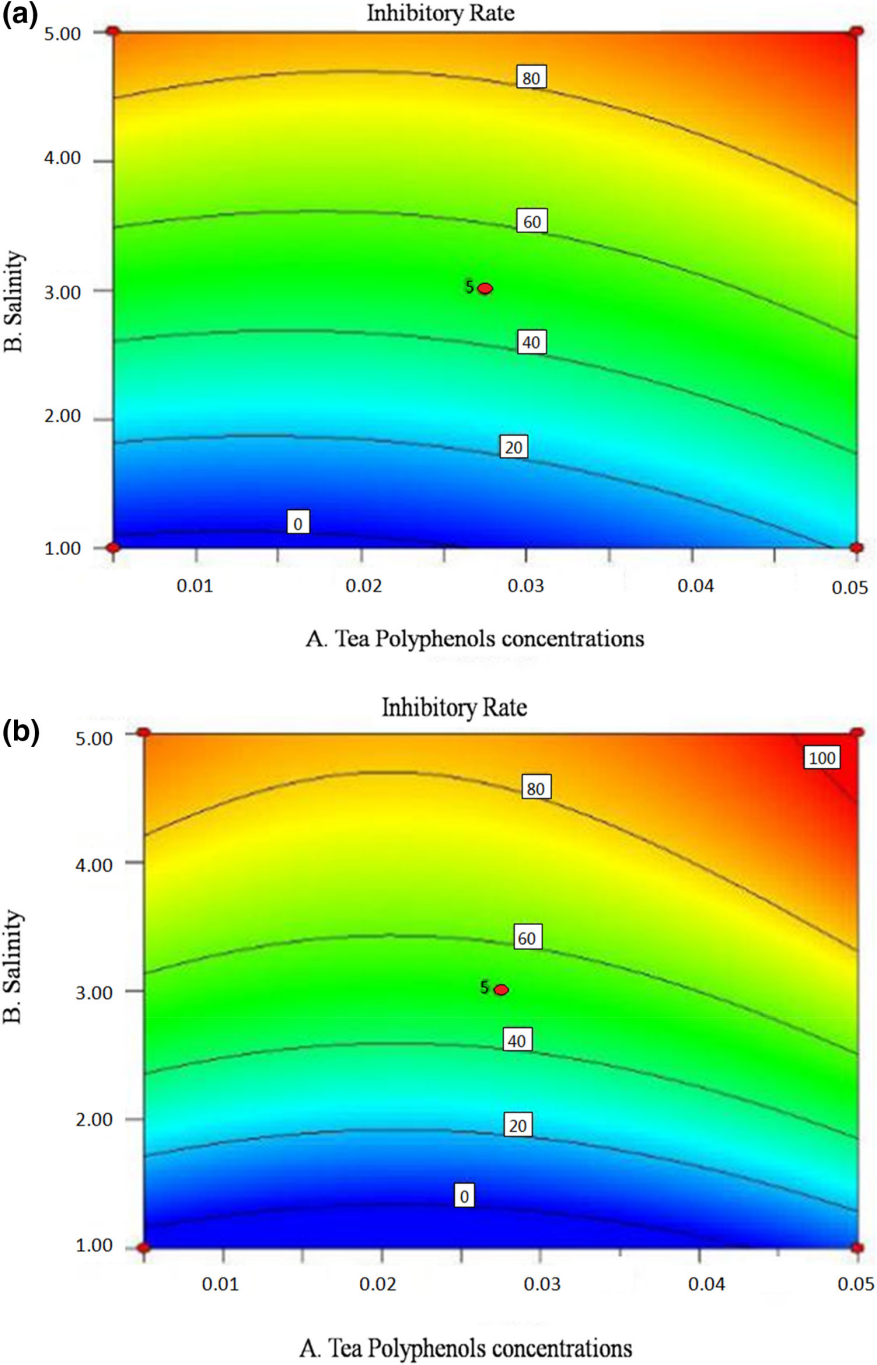
Contour map for the effects of tea polyphenol concentration and salinity on the inhibitory rate of SSOs. (a) Inhibitory rate of *Proteus vulgaris* under the interaction of tea polyphenol concentration and salinity; (b) Inhibitory rate of *Hafnia alvei* under the interaction of tea polyphenol concentration and salinity

**FIGURE 8:**
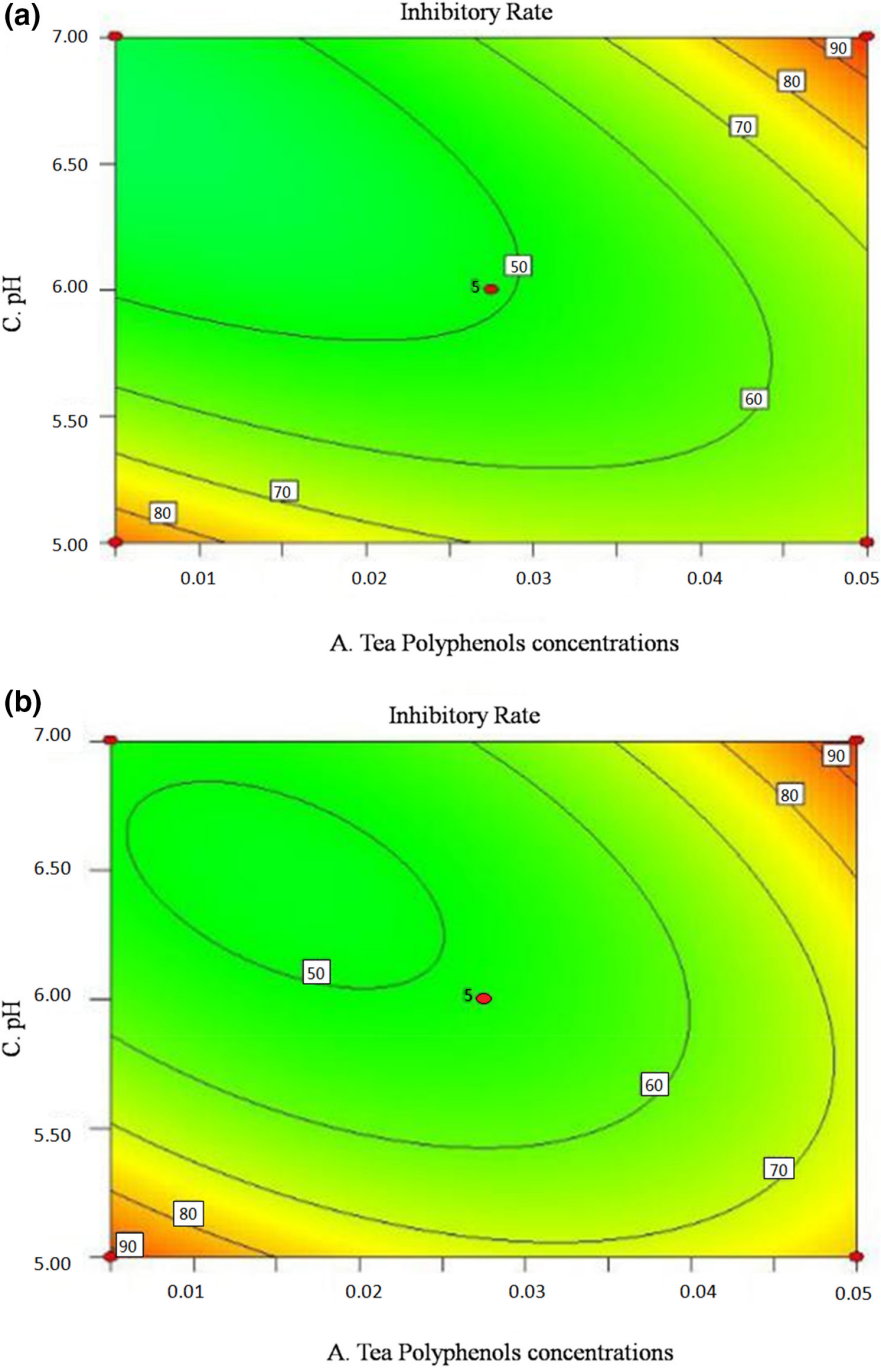
Contour map for the effects of tea polyphenol concentration and pH on the inhibitory rate of SSOs. (a) Inhibitory rate of *Proteus vulgaris* under the interaction of tea polyphenol concentration and pH; (b) Inhibitory rate of *Hafnia alvei* under the interaction of tea polyphenol concentration and pH

**FIGURE 9:**
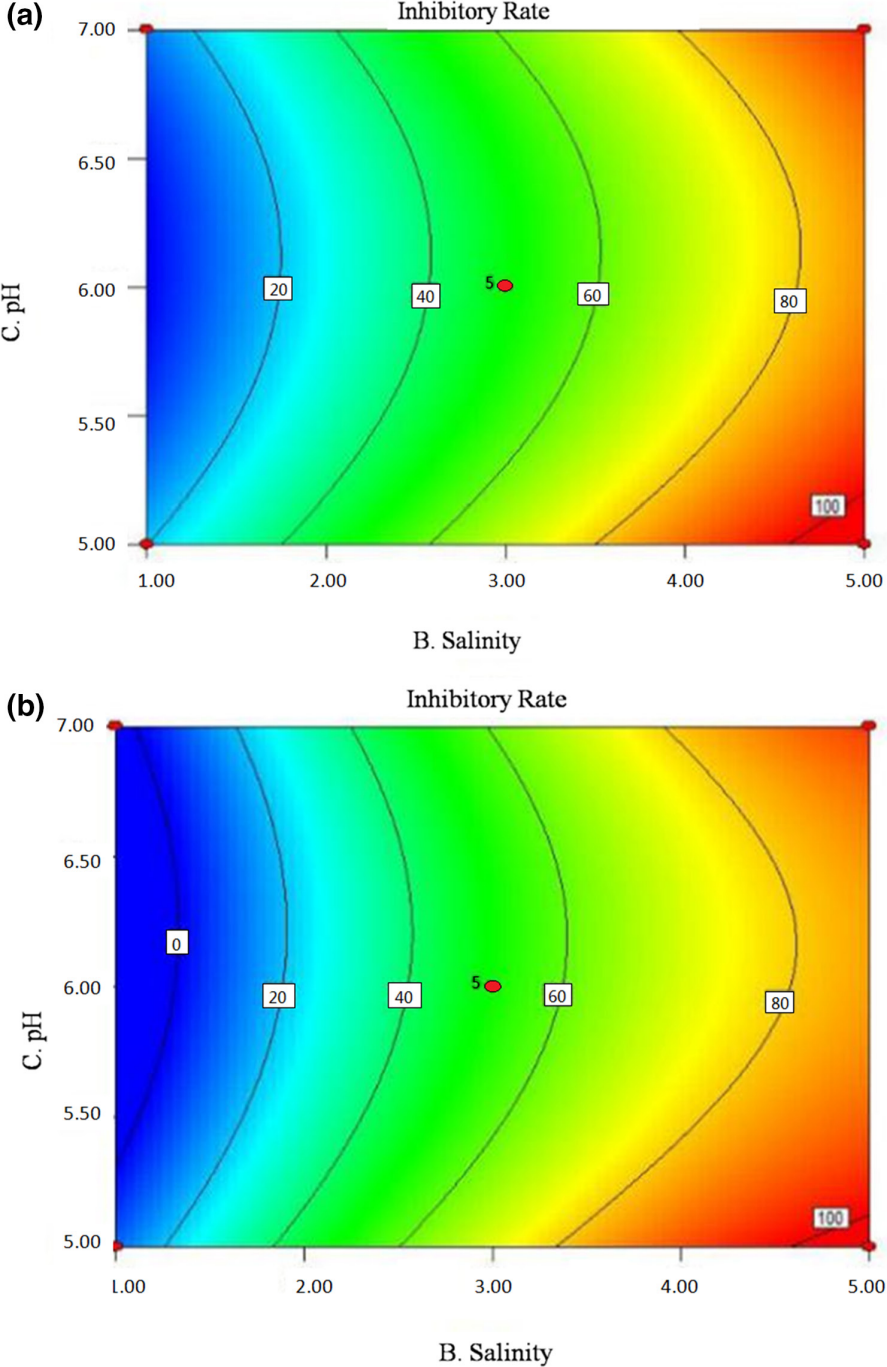
Contour map for the effects of salinity and pH on the inhibitory rate of SSOs. (a) Inhibitory rate of *Proteus vulgaris* under the interaction of salinity and pH; (b) Inhibitory rate of *Hafnia alvei* under the interaction of salinity and pH

To further investigate the interactions between the factors on the inhibitory effect, contour maps based on the fitting results are showed in Figures 7, 8, 9. Contour maps can directly reflect the relationships between the factors and response values, as well as the interaction effects between the factors (Liu et al., 2014). The steep slope of curves, elliptic contour, and dense contour lines in the graph indicated strong interactions; otherwise, the interactions were weak (Jia et al., 2010). In Figure 7, the gentle curve shows that the interaction between tea polyphenols concentration and salinity is weak. When the concentration of tea polyphenols was fixed, the inhibitory rate increased with the increase of salinity, but when the salinity was fixed, the rate barely changed with the increase of the tea polyphenols concentration. In Figure 8, the contour is elliptic, indicating that the interaction between tea polyphenols concentration and pH was strong, consistent with the regression analysis results of the previous model. Figure 9 shows that the curvature of the contour lines increases with the increase of salinity, indicating that the interaction between pH and salinity increases with the increase of salinity.

#### 3.2.3 Model optimization results and validation experiment

According to the results of RSM, the optimum antimicrobial conditions were tea polyphenols concentration 0.05%, salinity 3.46%, and pH 6.96 for *P*. *vulgaris*; tea polyphenols concentration 0.05%, salinity 3.45%, and pH 6.94 *for H. alvei*. Under this condition, a validation experiment was performed and result showed that the inhibitory rate was 100%, matching the predicted value, indicating that the response surface optimization results and the predicted model were reliable.

## 4 CONCLUSIONS

*Proteus vulgaris* and *H*. *alvei* were specific spoilage organisms isolated from the refrigerated lightly‐salted *large yellow croaker*. The results showed that tea polyphenols, salinity, and pH can inhibit the growth of both strains. When the concentration of tea polyphenols concentration and salinity increased to 0.05% and 5%, respectively, the strains did not grow. The results of Box–Behnken response surface showed that the optimum antibacterial parameters were as follows: tea polyphenols concentration 0.05%, salinity 3.46%, and pH 6.96 for *P*. *vulgaris*; tea polyphenols concentration 0.05%, salinity 3.45%, and pH 6.94 for *H*. *alvei*. Validation experiment was performed under the optimization conditions, and the inhibition rate were 100%. The research on the inhibitory effect of antibacterial factors on the spoilage bacteria of lightly‐salted large yellow croaker provides reference for the targeted inhibition of spoilage bacteria and the extension of shelf life of the product.

## ETHICAL APPROVAL

This study does not involve any human or animal testing.

## CONFLICT OF INTEREST

The authors declare that they do not have any conflict of interest.

## INFORMED CONSENT

Written informed consent was obtained from all study participants.
